# Visual duration aftereffect is position invariant

**DOI:** 10.3389/fpsyg.2015.01536

**Published:** 2015-10-09

**Authors:** Baolin Li, Xiangyong Yuan, Youguo Chen, Peiduo Liu, Xiting Huang

**Affiliations:** Key Laboratory of Cognition and Personality, Ministry of Education, Faculty of Psychology, Southwest UniversityChongqing, China

**Keywords:** vision, timing, adaptation, duration aftereffect, position

## Abstract

Adaptation to relatively long or short sensory events leads to a negative aftereffect, such that the durations of the subsequent events within a certain range appear to be contracted or expanded. The distortion in perceived duration is presumed to arise from the adaptation of duration detectors. Here, we focus on the positional sensitivity of those visual duration detectors by exploring whether the duration aftereffect may be constrained by the visual location of stimuli. We adopted two different paradigms, one that tests for transfer across visual hemifields, and the other that tests for simultaneous selectivity between visual hemifields. By employing these experimental designs, we show that the duration aftereffect strongly transfers across visual hemifields and is not contingent on them. The lack of position specificity suggests that duration detectors in the visual system may operate at a relatively later stage of sensory processing.

## Introduction

The brain performs actions that require precise timing on a daily basis, such as perception, speaking, or driving a car. Many studies have shown that perceived durations are distorted by recent sensory history (Johnston et al., 2006; Eagleman and Pariyadath, 2009; Heron et al., 2012a; Ortega et al., 2012; Zhou et al., 2014). A notable example of these misperceptions is the adaptation to relatively long or short sensory events, leading to a negative aftereffect such that the durations of the subsequent events within a certain range appear to be contracted or expanded (Walker et al., 1981; Becker and Rasmussen, 2007; Heron et al., 2012a). For example, after adaptation to a long duration of a repeating stimulus (640 ms), a subsequent stimulus of an intermediate duration (320 ms) appears shorter than it would otherwise, while after adaptation to a short duration repeating stimulus (160 ms), the duration of an intermediate stimulus (320 ms) tends to appear longer (Heron et al., 2012a). A neural adaptation model explains this aftereffect of perceived duration. This model proposes that there are time duration detectors in the brain, each of which responds selectively to a narrow range of stimuli durations centered on the detector’s preferred duration; moreover, the responses of these detectors diminishes with adaptation (Walker et al., 1981; Becker and Rasmussen, 2007; Heron et al., 2012a).

Perceiving time is an extremely complex psychological phenomenon, and its neural substrates remain elusive. Investigations of aftereffects provide crucial information about the mechanisms involved in processing specific visual attributes (Webster, 2011); duration aftereffects have been used in the cognitive neuroscience community to reveal the mechanisms of time perception. Previous duration adaptation studies have shown that the negative perceived duration aftereffect is bidirectional, modality specific, tuned around the adaptation duration (Walker et al., 1981; Becker and Rasmussen, 2007; Heron et al., 2012a), and precedes multisensory integration (Heron et al., 2013). According to these characteristics, Heron et al. (2012a, 2013) proposed that event duration is a low-level stimulus attribute, similar to visual spatial frequency or auditory pitch, and that duration selective neurons may operate at a relatively early stage of both visual and auditory sensory processing. However, our recent study demonstrates that the duration aftereffect is contingent on auditory pitch, but not on visual orientation of the stimulus. This result suggests the visual duration aftereffect from adaptation may originate at later stages of visual processing, or at least beyond primary visual cortex processing (Li et al., 2015). Hence, the neural locus of the visual duration adaptation remains to be elaborated.

There are several well-known visual perception adaptations and their corresponding aftereffects, such as the tilt (Gibson, 1937; Gibson and Radner, 1937; Magnussen and Kurtenbach, 1980), motion (Anstis et al., 1998; Hogendoorn and Verstraten, 2013), and face (Webster and Maclin, 1999; Webster et al., 2004) aftereffects. Compared to other visual perception adaptations, visual duration adaptation is poorly understood. One method to experimentally realize the visual duration adaptation is to test for the specificity of its aftereffect across various low-level properties, including size, orientation, and position. Furthermore, the viewer and the objects being viewed are both continuously moving in space, and thus the relative position of the visual object to the viewer is always changing. Therefore, position specificity, or invariance of the aftereffects, is a well-focused local feature related to adaptations, which may provide important clues regarding the level of visual processing at which the adaptations occur (Melcher, 2005). For example, the tilt aftereffect occurs only when the location of the test stimulus overlaps with the adapted spatial region (Gibson, 1937), while high-level aftereffects, such as face aftereffects, involve a position invariant mechanism (Leopold et al., 2001; Zimmer and Kovács, 2011). Thus, the goal of this study was to evaluate the positional sensitivity of the visual duration aftereffect.

Early visual cortex neurons are characterized by small retinotopically arranged receptive fields (Smith et al., 2001; Grill-Spector and Malach, 2004). If the visual duration adaptation begins in the early visual cortex (e.g., V1), then we would expect the visual duration aftereffect to show strong position specificity. Specifically, we expect not only the aftereffect constrained by the adapted visual hemifields of stimuli but also independent and significant aftereffects with opposite direction in the opposite visual hemifields following simultaneous adaptation to two opposite durations. Thus, in the present study, we designed two experiments to explore whether the duration aftereffect is position specific or position invariant. In the first experiment, observers adapted to a fixed duration defined by a visual stimulus (Gaussian blob) presented on one lateral side of the fixation cross, and were then tested with a range of randomly presented durations defined by the same visual stimulus on the left or right side of the fixation cross. This design allowed us to evaluate whether the duration aftereffect transfers across different visual hemifields. In the second experiment, observers simultaneously adapted to two different durations, defined by the same visual stimulus, presented alternately on the left or right side of the fixation cross, and were then tested as in the first experiment. This design allowed us to evaluate whether the duration aftereffect is contingent on visual hemifields.

## Materials and Methods

### Experiment 1

#### Participants

Eight individuals (four women, mean age = 22.25 years, *SD* = 1.58 years) participated in Experiment 1, including seven subjects naive to the experimental purpose and the first author. All reported normal or corrected to normal vision and hearing.

#### Apparatus and Stimuli

A Gaussian blob was used for the visual stimulus (*SD* = 0.53°, Michelson contrast = 0.74), which was presented on a 22″ CRT monitor (100 Hz refresh rate, 1024 × 768 pixels) with a gray background (9.0 cd/m^2^). The viewing distance was set to near 70 cm. The auditory stimulus was a white noise burst at ∼60 dB sound pressure level (SPL) presented through headphones with a 4 ms fade-in and fade-out. Stimuli presentation and data collection were implemented with computer programs designed with E-prime.

#### Procedures

**FIGURE 1:**
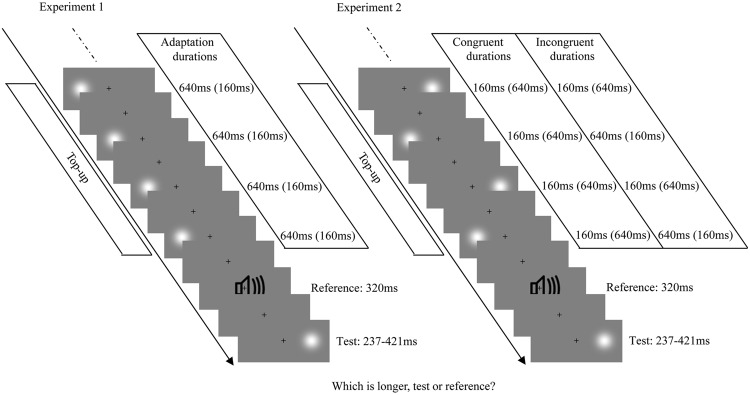
**Examples of each test-trial sequence from Experiments 1 **(Left)** and 2 **(Right)**.** The visual stimulus was a Gaussian blob and the auditory stimulus was a burst of white noise. Each test trial began with a top-up period, in which four top-up stimuli from the previously adaptation phase were presented. The top-up stimuli were always presented on one side of the fixation cross, with a fixed duration in Experiment 1, while they were alternately presented on the left and right sides of the fixation cross, with congruent or incongruent durations in Experiment 2. After the top-up period, the reference and the test stimulus (randomly presented on the left or right side of the fixation cross) were successively presented.

The procedures were similar to the main visual adaptation experiments of Heron et al. (2012a). At the beginning of the formal experiment, the adaptation stimulus was presented 100 times, followed by a further four top-up stimuli after a 2000-ms pause. During the adaptation phase and the top-up period, the Gaussian blobs with fixed duration (160 or 640 ms) were presented on one side of a central fixation cross (0.4° × 0.4°; centered 10° to the left or right of the fixation). Subsequently, white noise lasting 320 ms was presented as the reference cue. Next, the test stimulus (a Gaussian blob) was presented with a duration that varied in seven logarithmically spaced steps, from 237 to 421 ms, which were randomly interleaved using a method of constant stimuli. The test stimulus could be randomly located at either 10° to left or right of the fixation cross. That is, the position of the adaptation stimulus and test stimulus could either be same (overlapping) or different (in the opposite hemifields). Observers were asked to make an unspeeded, two-alternative forced-choice duration discrimination judgment by pressing the “F” or “J” buttons on the computer keyboard after the test stimulus had disappeared (buttons were counterbalanced between participants). Once the response occurred, the next top-up-test cycle was automatically triggered after a randomly jittered pause between 500 and 1000 ms (the same range as the inter-stimulus interval between adaptation, top-up, reference, and test stimuli). Observers were strictly instructed to keep their eyes on the fixation cross and attend to the duration of each stimulus during the entire experiment, but were not asked to make a perceptual judgment until the test stimulus was presented (see **Figure 1**, left). There were four adaptation conditions: “LS,” left short (160 ms); “LL,” left long (640 ms); “RS,” right short (160 ms); “RL,” right long (640 ms). For each adaptation condition, observers completed two blocks of 70 test trials with five trials for each of the two test locations at each of the seven possible durations. Each observer completed four adaptation conditions in a single day, which were repeated over 2 days, resulting in a total of 560 trials. Both the order of trials in a given block and the order of blocks each day were selected randomly. The daily experiment began with practice trials until the participant was comfortable in performing the duration discrimination judgment.

#### Results

The proportion of “longer” responses to test stimuli for each condition (4 adaptations × 2 test locations) was plotted as a function of test duration and fitted with a logistic function of the form:

$$y=\frac{1}{1+e^{-\frac{\left(X\;-\;X0\right)}{b}}}\;\;\;\;(\mathrm{see}\;Figure\;2\;\mathrm{for}\;\mathrm{overall}\;\mathrm{data}).$$

**FIGURE 2:**
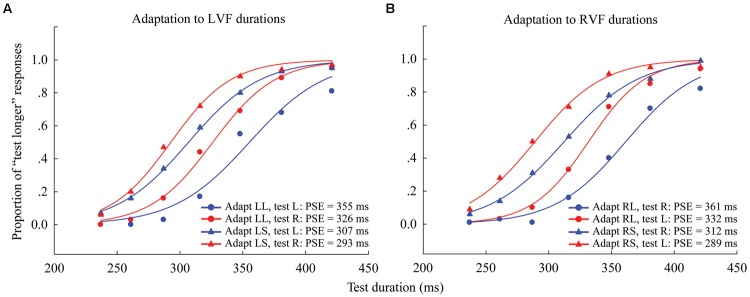
**Psychometric functions for eight observers showing the proportion of “longer” responses to test stimuli as a function of test duration in Experiment 1. (A)** Adaptation to left visual field (LVF) durations where the left adaptation duration was long (“LL,” circle symbols) or short (“LS,” triangle symbols), and the test stimulus was either located to the left (blue line, same condition) or right (red line, different condition). **(B)** Adaptation to RVF durations, where the right adaptation duration was long (“RL,” circle symbols) or short (“RS,” triangle symbols), and the test stimulus was located to either the left (red line, different condition) or right (blue line, same condition). The point of subjective equality (PSE), as indicated by the physical test duration corresponding to 50% “test longer” responses, for each condition is shown at the lower right.

Where X0 is the test duration value corresponding to the point of subjective equality (PSE; 50% response level on the psychometric function) and *b* provides an estimate of the duration discrimination threshold (approximately half the offset between the 27 and 73% response levels). The PSE values were obtained for all observers in all of the conditions. In order to compare the PSE values across conditions, the “Aftereffect magnitude” was calculated as the arithmetic difference between the PSE values for each adapting polarity and spatial location:

$$\mathrm{Aftereffect}\;\mathrm{magnitude}=({\mathrm{PSE}}_{\mathrm{adapt}\;\;L})-({\mathrm{PSE}}_{\mathrm{adapt}\;\;S}).$$

For example, when the adaptor in the left visual field (LVF), the aftereffect magnitude in the same (or different) position would be the arithmetic difference between the PSE values of the left (or right) test stimulus in the “LL” and “LS” adaptation conditions. When the adaptor in the right visual field (RVF), the aftereffect magnitude in the same (or different) position would be the arithmetic difference between the PSE values of the right (or left) test stimulus in the “RL” and “RS” adaptation conditions. In this way, the aftereffect magnitudes were obtained for each observer (see Supplementary Table S1).

**FIGURE 3:**
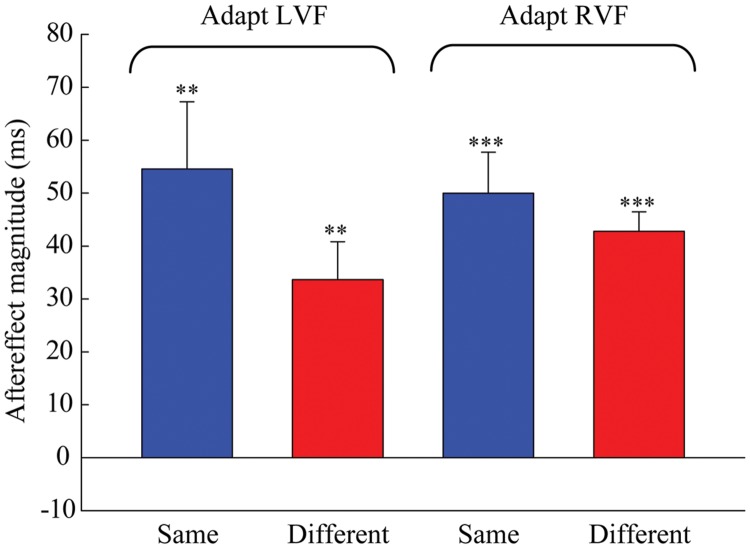
**Aftereffect magnitudes averaged across the eight observers for each condition in Experiment 1.** The height of the bars represents the arithmetic difference between the PSE values for each adapting polarity and spatial location in Experiment 1. The same conditions (blue bar) denote situations where the adaptation and test stimuli were presented in the same hemifield. The different conditions (red bar) denote situations where the adaptation and test stimuli were presented in the opposite hemifields. Error bars represent the SEM across observers. (^∗∗^*p* < 0.01; ^∗∗∗^*p* < 0.001)

Next, one-sample two-tailed *t*-tests showed that the aftereffect magnitudes were significantly larger than zero when the adaptor was in the LVF [same: mean = 54.552, *SEM* = 12.717, *t*(7) = 4.29, *p* = 0.004; different: mean = 33.685, *SEM* = 7.166, *t*(7) = 4.7, *p* = 0.002] as well as in the RVF [same: mean = 49.978, *SEM* = 7.769, *t*(7) = 6.433, *p* < 0.001; different: mean = 42.808, *SEM* = 3.686, *t*(7) = 11.615, *p* < 0.001] (see **Figure 3**). A 2 × 2 repeated-measures ANOVA (within-subjects design) with two levels of adaptation field (LVF, RVF) and two levels of position (same, different) was applied to the aftereffect magnitudes. The ANOVA revealed that both the main effect of adaptation field and the main effect of position were not significant [*F*(1,7) = 0.097, *p* = 0.765; *F*(1,7) = 1.938, *p* = 0.207], and that their interaction was marginally significant [*F*(1,7) = 5.54, *p* = 0.051]. Furthermore, the simple effect analysis showed that the aftereffect magnitudes between the same and different conditions had no significant differences, either in the LVF [*F*(1,7) = 2.78, *p* = 0.139] or the RVF [*F*(1,7) = 0.81, *p* = 0.397] adaptation condition. These results suggest the aftereffect of perceived duration can transfer across visual hemifields.

### Experiment 2

#### Participants

Experiment 2 included a separate set of five subjects naive to the experimental conditions and the first author (three women, mean age = 20.5 years, *SD* = 1.87 years). All reported normal or corrected to normal vision and hearing.

#### Design and Procedures

The apparatus, stimuli, and procedures were similar to those used in Experiment 1, except for the position of the stimulus in the adaptation phase and top-up period. During Experiment 2’s adaptation phase and top-up period, Gaussian blobs were presented with congruent or incongruent durations, and alternated between locations, positioned either 10° to the left or to the right of the fixation cross (see **Figure 1**, right). There were four resulting adaptation conditions: “LSRS,” both left and right short (160 ms); “LLRL,” both left and right long (640 ms); “LSRL,” left short (160 ms) and right long (640 ms); and “LLRS,” left long (640 ms) and right short (160 ms). For each adaptation condition, observers completed four blocks of 70 test trials with five trials for each of the two test locations at each of the seven possible durations. Each subject completed four adaptation conditions in a single day, which were repeated over four days, resulting in a total of 1120 trials. The positions of the starting stimulus during the adaptation phase (left first or right first) were counterbalanced: half the subjects observed the sequence ABBA across 4 days, while the other half observed BAAB (A and B represent left first and right first, respectively).

#### Results

**FIGURE 4:**
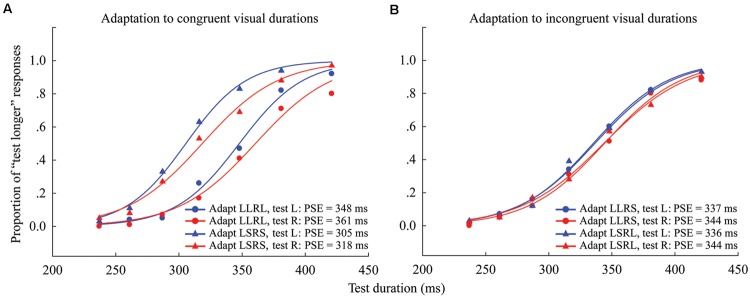
**Psychometric functions for six observers showing the proportion of “longer” responses to test stimuli as a function of test duration in Experiment 2. (A)** Adaptation to congruent visual durations where both left and right adaptation durations were long (“LLRL,” circle symbols) or short (“LSRS,” triangle symbols), and the test stimulus was either located to the left (blue line) or right (red line) of the fixation cross. **(B)** Adaptation to incongruent visual durations where the left adaptation duration was long and the right adaptation duration was short (“LLRS,” circle symbols), or the left adaptation duration was short and the right adaptation duration was long (“LSRL,” triangle symbols), and the test stimulus was either located at left (blue line) or right (red line). The PSE for each condition is shown at the lower right.

For each observer, the PSE was calculated for each condition (4 adaptations × 2 test locations) as in Experiment 1 (see **Figure 4** for overall data). The “Aftereffect magnitude” was also calculated as the arithmetic difference between the PSE values for each adapting polarity and spatial location (see Supplementary Table S2). For example, in the congruent adaptation condition, for the left (or right) location, the aftereffect magnitude was the arithmetic difference between the left (or right) test stimulus PSE values in the “LLRL” and “LSRS” conditions. In the incongruent adaptation condition, for the left (or right) location, the aftereffect magnitude was the arithmetic difference between the PSE values of the left (or right) test stimulus in the “LLRS” (“LSRL”) and “LSRL” (“LLRS”) conditions.

**FIGURE 5:**
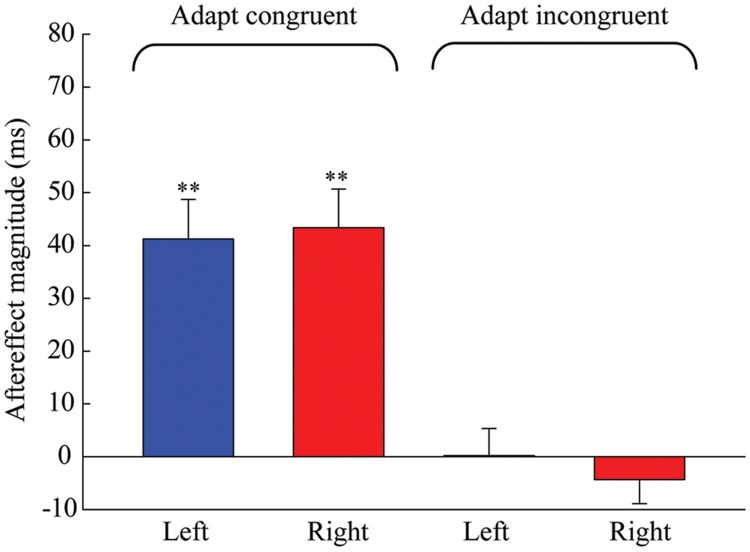
**Aftereffect magnitudes averaged across the six observers for each condition in Experiment 2.** Blue and red bars represent the conditions where the locations of test stimuli were at left and right, respectively. Error bars represent the SEM across observers. (^∗∗^*p* < 0.01).

The results of the one-sample 2-tailed *t*-tests showed that the aftereffect magnitudes of both the left [mean = 41.259, *SEM* = 7.489, *t*(5) = 5.51, *p* = 0.003] and right locations [mean = 43.379, *SEM* = 7.316, *t*(5) = 5.929, *p* = 0.002] were significantly larger than zero in the congruent adaptation condition. However, in the incongruent adaptation condition, there was no significant difference from zero for the aftereffect magnitude in either the left [mean = 0.227, *SEM* = 5.132, *t*(5) = 0.044, *p* = 0.966] or right location [mean = –4.313, *SEM* = 4.575, *t*(5) = –0.943, *p* = 0.389] (see **Figure 5**). A 2 × 2 repeated-measures ANOVA (within-subjects design) with two levels of adaptation congruency (congruent, incongruent) and two levels of test location (left, right) was performed on the aftereffect magnitudes. The main effect of adaptation congruency was significant [*F*(1,5) = 30.434, *p* = 0.003], showing that the aftereffect magnitude in the congruent adaptation condition was significantly larger than the aftereffect magnitude in the incongruent adaptation condition. However, the main effect of the test location [*F*(1,5) = 0.049, *p* = 0.833] and the interaction [*F*(1,5) = 0.715, *p* = 0.436] were not significant. These results suggest that the duration aftereffect is not contingent on visual hemifields.

We also compared the aftereffect magnitudes between the same condition in Experiment 1 and the congruent adaptation condition in Experiment 2 to determine whether the variable location of the adaptation stimuli can affect the aftereffect magnitude. A 2 × 2 repeated-measures ANOVA (mixed-subject design with adaptation type as the between-subjects factor) with two levels of adaptation type (asynchronous, simultaneous) and two levels of test location (left, right) was performed on the aftereffect magnitudes. The ANOVA revealed that the main effect of adaptation type [*F*(1,12) = 0.602, *p* = 0.453], the main effect of test location [*F*(1,12) = 0.056, *p* = 0.817], and their interaction [*F*(1,12) = 0.417, *p* = 0.531] were not significant. The lack of difference between the adaptation types suggests that the stimulus location in the adaptation phase has no effect on the aftereffect magnitude.

## Discussion

In the current study, we investigated the positional sensitivity of the visual duration aftereffect. In the first experiment, our results showed that the aftereffect of the perceived duration transfers across different visual hemifields, as there were clear aftereffects in both the same-side and different-side conditions. In the second experiment, we found that aftereffects disappeared in the incongruent adaptation condition, implying that the perceived duration aftereffect is not contingent on visual hemifields. Additionally, when comparing the aftereffect magnitudes from Experiments 1 and 2, we found that the variable location of the adaptation stimuli in Experiment 2 did not influence the aftereffect magnitude. Taken together, these findings suggest that the visual duration aftereffect is position invariant.

The results of the current study appear to contradict those of earlier studies that show the importance of spatial alignment on temporal-aftereffects. For example, studies have shown that the duration-compression aftereffect, which can be induced by adaptation to a flickering (e.g., 20 Hz) visual stimulus and subsequent testing with a visual stimulus flickering at a different frequency (e.g., 10 Hz), is position specific (Ayhan et al., 2009; Bruno et al., 2010; Burr et al., 2011). Additionally, a recent study also showed that the visual duration-compression effect induced by a prime is related to the spatial proximity to that prime (Zhou et al., 2014). These results suggest that a position specific mechanism is involved in time perception. However, these duration-compression effects do not use any repeated presentation of duration as an adaptor. In our opinion, they are different from the duration aftereffect induced by adaptation to the duration length itself. Our findings suggest that a position invariant mechanism is also involved in time perception. This opinion has been expressed in other studies. For example, Nagarajan et al. (1998) found a learning effect in somatosensory interval discrimination that generalizes completely across untrained skin locations, even those in the contralateral hand. Additionally, studies have shown that the audiovisual temporal recalibration is not constrained by spatial location (Keetels and Vroomen, 2007; Roseboom and Arnold, 2011; but see Heron et al., 2012b). Given the dynamic environment in which humans exist, the position invariant mechanism in time perception may have functional and ecological significance. For example, consider a game of table tennis where the ball moves from one side to the other side; the perceived duration of the ball is continuous and stable even when we keep our head and eyes fixed. The position invariant time perception is certainly advantageous in forming a stable representation of the external world.

In our study, we found that the observed duration aftereffect transfers to the opposite hemifields, which is similar to the high-level face aftereffects (Kovács et al., 2005, 2007). This result suggests that the visual duration aftereffect may be the product of adaptation from high-level neurons that have large visual receptive fields, which cover both sides of the fixation and extend into the ipsilateral visual field. This information, combined with findings from our previous study showing that the duration aftereffect is not contingent on visual orientation (Li et al., 2015), suggests that visual event duration is a high-level stimulus attribute and that the duration detectors in the visual system may be involved at a later stage of sensory processing. Consistent with this idea, electrophysiological studies have found visual duration-sensitive neurons located at much later neural loci, both for supra-second timing (such as the prefrontal/frontal cortex, Genovesio et al., 2006, 2009) and sub-second timing (such as the posterior parietal cortex, Leon and Shadlen, 2003; but see Duysens et al., 1996).

However, our results do not exclude the existence of neurons located at the early stage of visual processing that are sensitive to duration information. Recent studies of temporal aftereffects induced by adaptation to non-duration information have suggested that neurons at the early stages of the visual system, including magnocellular neurons (Johnston et al., 2006, 2008; Ayhan et al., 2009, 2011; Bruno et al., 2010) and V1 neurons (Ortega et al., 2012; Zhou et al., 2014), are involved in visual time perception. Although the traditional view toward sub-second temporal processing assumes that there is a centralized mechanism responsible for timing, such as the internal clock model (Treisman, 1963; Treisman et al., 1990, 1994), accumulating evidence has demonstrated the existence of multiple timing mechanisms in the brain (Ivry and Schlerf, 2008; Merchant et al., 2013). Given these facts, we think that both low-level and high-level timers coexist in the visual system.

## Conclusion

In the present study, we used two experiments to investigate the positional sensitivity of the visual duration aftereffect. We found that the perceived duration aftereffect transfers strongly across visual hemifields and is not contingent on those visual hemifields. These results suggest that the perceived visual duration aftereffect is position invariant. The lack of spatial specificity suggests duration detectors in the visual system may operate at a relatively later stage of sensory processing.

## Ethics Statement

This study was approved by the local ethics committee of the Southwest University of China. The experiments were conducted in accordance with the Declaration of Helsinki. All naive participants gave written informed consent prior to the experiments and were paid for their participation.

## Conflict of Interest Statement

The authors declare that the research was conducted in the absence of any commercial or financial relationships that could be construed as a potential conflict of interest.
