# Supplementary material for: Visual duration aftereffect is position invariant
Source: Front Psychol. 2015 Oct 9;6:1536. doi: 10.3389/fpsyg.2015.01536 (PMC4598571; doi:10.3389/fpsyg.2015.01536)
Supplement: Supplementary file 1 [file Table_1.PDF]

Supplementary Results

Table S1. Aftereffect magnitude for each participant/condition in Experiment 1

Experiment 2a: Participants	Adapt LVF		Adapt RVF	
	Same	Different	Same	Different
1	70.814	51.644	79.276	60.257
2	28.559	23.008	32.754	44.019
3	36.387	7.567	60.169	50.324
4	12.674	22.777	28.227	45.780
5	126.077	27.285	84.194	31.997
6	78.684	61.001	41.262	43.601
7	41.519	58.251	31.362	39.909
8	41.703	17.945	42.576	26.574

Table S2. Aftereffect magnitude for each participant/condition in Experiment 2

Experiment 2b: Participants	Adapt congruent		Adapt incongruent	
	Left	Right	Left	Right
1	30.824	36.967	-15.651	2.289
2	15.107	30.496	-10.015	10.555
3	68.252	76.384	12.435	-22.491
4	43.769	35.690	16.632	-1.291
5	36.755	29.595	1.415	-8.846
6	52.848	51.140	-3.451	-6.092
